# Supplementary material for: Explaining placebo effects in an online survey study: Does ‘Pavlov’ ring a bell?
Source: PLoS One. 2021 Mar 11;16(3):e0247103. doi: 10.1371/journal.pone.0247103 (PMC7951811; doi:10.1371/journal.pone.0247103)
Supplement: S1 File — (PDF) [file pone.0247103.s001.pdf]

APPENDIX I. Overview of placebo explanations based on their underlying mechanisms

**Mechanism**	**Placebo explanation**	**Explanation obtained from**
Conditioning	"Learning does not only happen in the brain, but also in the body, even when you are not aware of this. Our body can learn from taking medication; it remembers what happens and what chemicals are produced. This is called conditioning. In this placebo treatment, we will make use of the body's learned response to reduce pain."	[20, 22, 24, 19]
Positive expectations	"You can have expectations about a treatment and its outcomes. If you have positive expectations, this may cause for a more positive treatment outcome and it may also affect your pain experience in this present study. You may not only feel better from the treatment itself, but also because you expect to feel better."	[20,22, 24, 19, 16, 26]
Brain	"When you have positive expectations, the brain produces chemicals. These chemical substances are called neurotransmitters and can make you feel better. Placebos can also trigger the release of neurotransmitters. In this treatment, we will be making use of this chemical process to reduce pain."	[20, 26]
Mind and body	"This treatment is based on the interaction between **mind and body**. The mind plays a big role in healing. The placebo effect is an example of this, because it shows that treatments without active medication (placebo pills, like sugar pills) can also evoke a physical reaction."	[21, 19]
Social learning	"Placebo treatment make use of the **experiences** that others have with treatments. Seeing or hearing others benefit from treatment may cause you to feel better after this treatment as well."	Not used before
Trust	"A good relationship with your doctor can have a positive impact on treatment outcomes. For example, it is important that the doctor makes you feel that he **trusts** in the prescribed treatment. Placebo treatments are based on the importance of this relationship of trust."	Not used before
Neutral	"This treatment is based on the placebo effect. This treatment may help you, but we are still researching how this exactly works. We can find out if this works for you too."	Not used before
Transparency	"**Honesty** is the most important factor in this treatment. Previously, it was thought that deception was necessary in order for a placebo effect to occur. However, research has demonstrated that placebo effects are also present when people know they are taking a placebo. We would like to apply this in this treatment."	[20], [26]

Note. All explanations are translated from the original Dutch survey. Participants were instructed to imagine that a doctor prescribed a placebo treatment with the abovementioned explanations. The explanations were pictured in a text box with an animated picture of a doctor.
