# Supplementary material for: Explaining placebo effects in an online survey study: Does ‘Pavlov’ ring a bell?
Source: PLoS One. 2021 Mar 11;16(3):e0247103. doi: 10.1371/journal.pone.0247103 (PMC7951811; doi:10.1371/journal.pone.0247103)
Supplement: S2 File — (PDF) [file pone.0247103.s002.pdf]

APPENDIX II

Comparison of sample within 1.5 IQR and sample exceeding 1.5 IQR *(N*=377*)*

	**Preference scores**			**Perceived efficacy**			**Willingness to participate**		
	**IQR<1.5** (N=348)	**IQR>1.5** (N=29)	***p***	**IQR<1.5** (N=346)	**IQR>1.5** (N=31)	***p***	**IQR<1.5** (N=351	**IQR>1.5** (N=26)	***p***
**Mean (SD)**	5.99 (.06)	3.90 (.21)	**<*.001***	.00[a] (.07)	.64[a] (.22)	**<*.01***	-.23[a] (.07)	3.58[a] (.24)	**<*.001***
**Age[b]**									
**Median (IQR)**	23 (20-28)	23 (21-26.5)	.745	23 (20-28)	23 (21-28)	.885	23 (20-28)	23 (21-29)	.712
**Sex[c]**									
**Female**	221 (63.5%)	19 (65.5%)	1.00	224 (64.7%)	16 (51.6%)	.173	225 (64.1%)	15 (57.7%)	.531
**Male**	127 (36.5%)	10 (34.5%)		122 (35.3%)	15 (48.4%)		126 (35.9%)	11 (42.3%)	
**Education[c]**									
**Low**	38 (10.9%)	1 (3.4%)	.339	37 (10.7%)	2 (6.5%)	.757	37 (10.5%)	2 (7.7%)	1.00
**High**	310 (89.1%)	28 (96.6%)		309 (89.3%)	29 (93.5%)		314 (89.5%)	24 (92.3%)	
**LOT-R[d]**									
**Mean (SD)**	20.28 (3.61)	19.83 (4.33)	.525	20.35 (3.62)	19.03 (4.00)	.054	20.27 (3.63)	19.96 (4.15)	.684
**STAI[d]**									
**Mean (SD)**	47.14 (4.38)	45.83 (5.46)	.128	46.96 (4.38)	48.00 (5.42)	.212	46.58 (4.36)	49.65 (5.16)	**<*.01***
**GAMQ[d]**									
**Mean (SD)**	38.63 (3.19)	38.31 (2.95)	.609	38.50 (3.15)	39.74 (3.19)	**<*.05***	38.49 (3.18)	40.04 (2.69)	**<*.05***
**NEO[d]**									
**Mean (SD)**	18.72 (4.90)	18.48 (5.15)	.802	18.83 (4.89)	17.29 (5.10)	.095	18.75 (4.93)	18.04 (4.69)	.476
**PlaceboQuiz[d]**									
**Mean (SD)**	81.86 (12.88)	80.79 (10.30)	.664	82.23 (12.66)	76.73 (12.09)	**<.05**	81.87 (12.73)	80.49 (12.38)	.595
**Medication use[c]**									
**Yes***	220 (63.2%)	21 (72.4%)	.422	222 (64.2%)	19 (61.3%)	.846	225 (64.1%)	16 (61.5%)	
**No**	128 (36.8%)	8 (27.6%)		127 (35.8%)	12 (38.7%)		126 (35.9%)	10 (38.5%)	.834

*Differences between data with and without outliers were found on the predictor variables general attitudes towards medication, trait anxiety and PlaceboQuiz, but not consistent on all three scores. Note: *Medication use: pain analgesia (57.5%), birth control (15.8%), allergies/asthma/eczema (7.9%), and other (18,8%, i.e. thyroid medication, insulin, antidepressants and ADHD/ADD medication) .[a]Transformed scores (based on component loadings from CATPCA), [b]Mann-Whitney U Test, ***Chi Square Test, ****Analysis of Variance*
