# Supplementary material for: Explaining placebo effects in an online survey study: Does ‘Pavlov’ ring a bell?
Source: PLoS One. 2021 Mar 11;16(3):e0247103. doi: 10.1371/journal.pone.0247103 (PMC7951811; doi:10.1371/journal.pone.0247103)
Supplement: S3 File — (PDF) [file pone.0247103.s003.pdf]

APPENDIX III: Additional analyses based on component scores for perceived efficacy and willingness to participate outcomes.

Perceived efficacy

Explanation comparisons. Based on the two-dimensional structure of the CATPCA, the two components were entered as within-subject factors in a repeated measures ANCOVA, and variables age, gender, education level, dispositional optimism, trait anxiety, neuroticism, placebo knowledge and attitudes towards medication were entered as potential predictors. The results indicated that the model was significant, indicating that the explanations were perceived as effective $F(1,367) = 9{,}173$, $p < 0.01$. Bonferroni-corrected post-hoc tests for pairwise comparisons indicated that both components were perceived equally effective.

Predictors for perceived efficacy scores. Perceived efficacy was associated with the predictors optimism ($F(1,367) = 9{,}020$, $p < 0.01$, $\eta p^2 = .024$), trait anxiety ($F(1,367) = 7{,}788$, $p < .01$, $\eta p^2 = .021$), placebo knowledge ($F(1,367) = 5{,}446$, $p < 0.05$, $\eta p^2 = .015$), and education ($F(1,367) = 7{,}788$, $p < 0.01$, $\eta p^2 = .024$) on perceived efficacy for both components (see Figure 7 for all differential effects of the significant predictors).

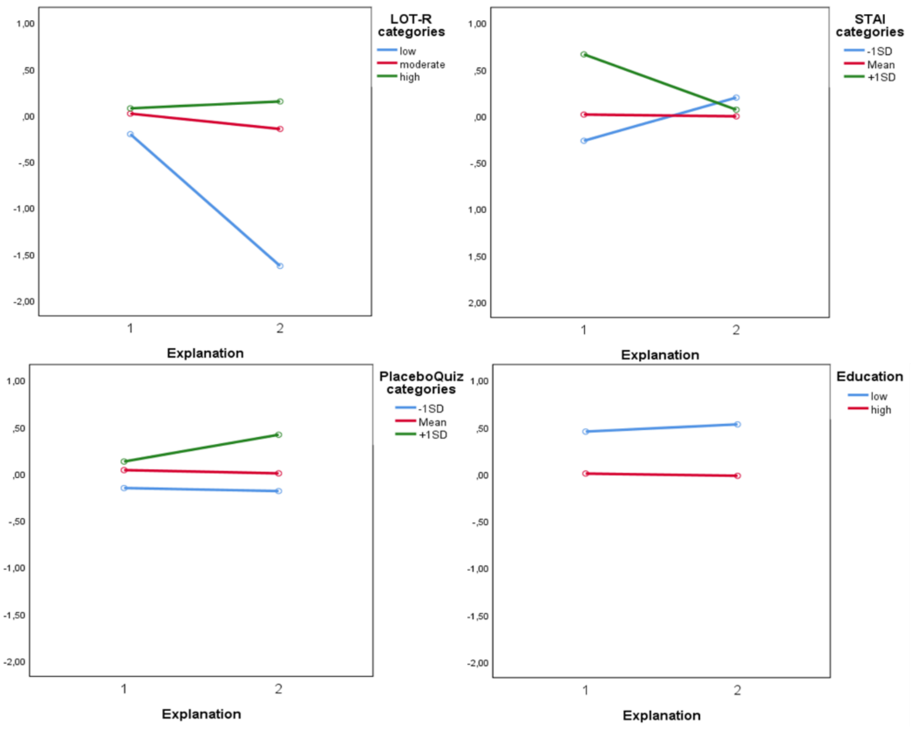

Figure 7. Differential effects of significant predictors optimism (LOT-R), trait anxiety (STAI), placebo knowledge, and education scores for perceived efficacy of placebo explanations. All 8 explanations were reduced to two components: component 1 (expectations, brain mechanisms, mind and body, social learning and trust) and component 2 (conditioning, transparency and the neutral explanation). Values on the *y*-axis represent respective component loadings.

Willingness to participate

Explanation comparisons. To compare willingness to participate scores, the procedure from the perceived efficacy scale was repeated with the two new components as time points for willingness to participate. The model indicated that there was no difference in willingness to participate between the components of different explanations.

Predictors for willingness to participate. Trait anxiety (F(1,368) = 12,672, p < .01, ηp^2 = .033) and general attitudes towards medication (F(1,368) = 4.540, p < .05, ηp^2 = .012) were significant predictors for willingness to participate, indicating that participants with higher anxiety scores were more willing to participate based on the first component (expectations, mind and body, trust, conditioning and brain mechanisms) and individuals with low trait anxiety scores were more willing to participate based on the second component (social learning, transparency and the neutral explanation). This was also the case for participants with more positive attitudes towards medication, who were more willing to participate based on component 1, and participants with low general attitudes towards medication scores on component 2 (see Figure 8).

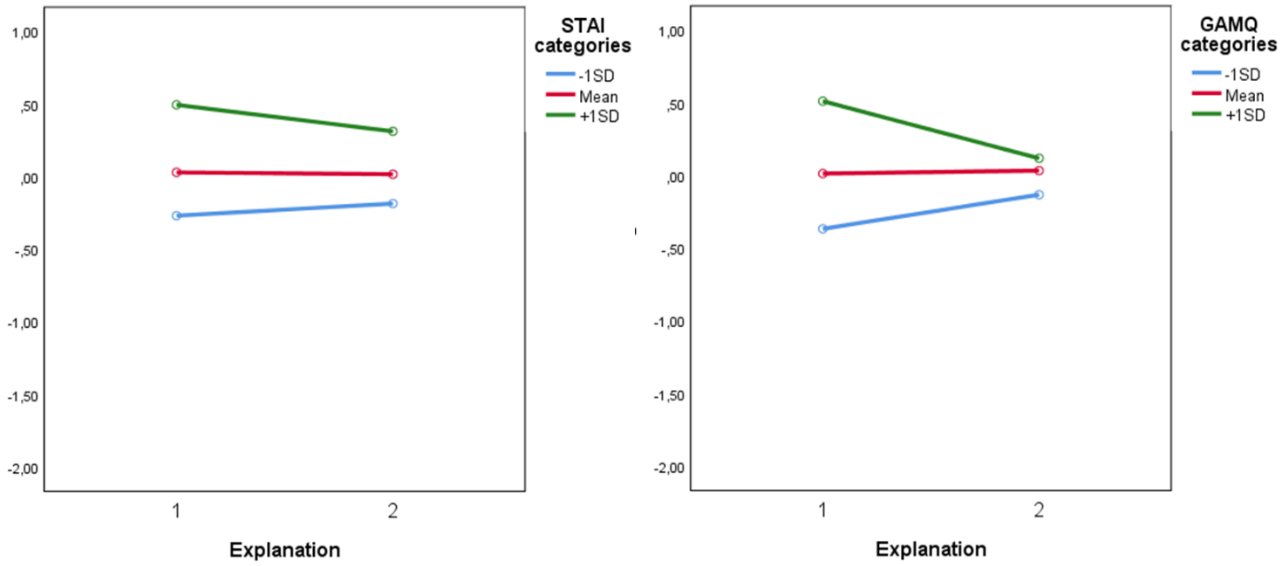

Figure 8. Differential effects of significant predictors trait anxiety (STAI) and general attitudes towards medication (GAMQ). All 8 explanations were reduced to two components: component 1 (expectations, brain mechanisms, mind and body, conditioning and trust) and component 2 (social learning, transparency and the neutral explanation). Values on the y-axis represent component loadings.
