# Supplementary material for: Explaining placebo effects in an online survey study: Does ‘Pavlov’ ring a bell?
Source: PLoS One. 2021 Mar 11;16(3):e0247103. doi: 10.1371/journal.pone.0247103 (PMC7951811; doi:10.1371/journal.pone.0247103)
Supplement: S4 File — (PDF) [file pone.0247103.s004.pdf]

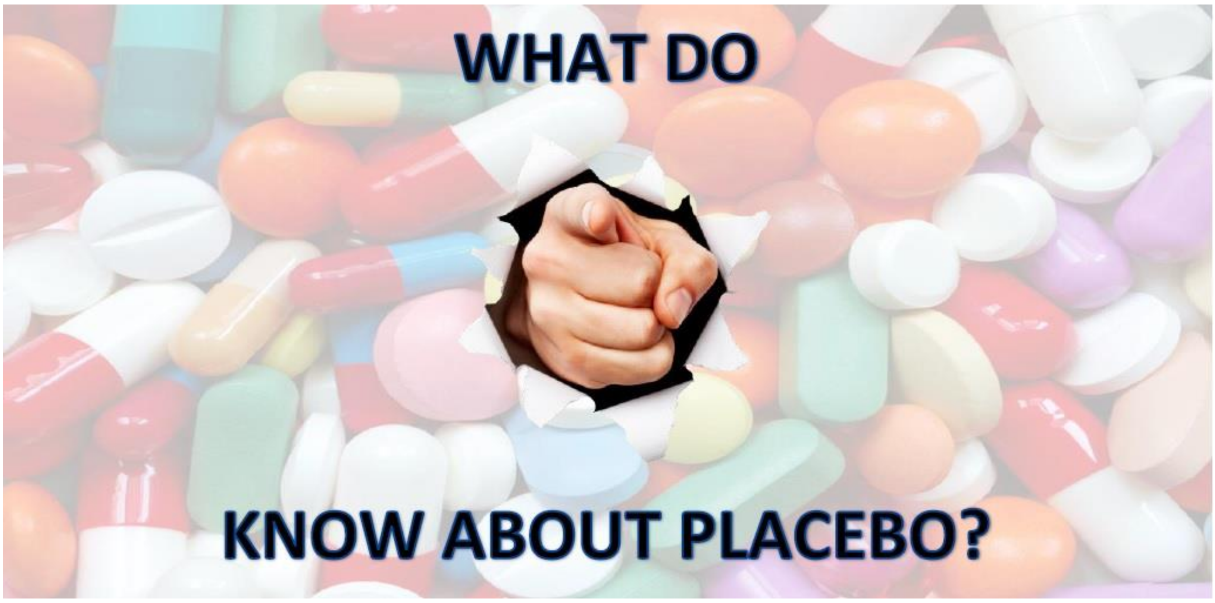


**Placebo's**

Placebo’s worden vaak gebruikt in wetenschappelijk onderzoek. Dit zijn behandelingen die geen actieve bestanddelen bevatten. Toch kunnen mensen zich na het krijgen van een placebo beter voelen. Dit wordt het placebo-effect genoemd.

U kunt alle vragen beantwoorden vanuit uw eigen perspectief en op basis van wat u zelf weet over placebo’s. Het is belangrijk dat u hierin zo eerlijk mogelijk bent. U kunt geen goede of foute antwoorden geven, wij zijn enkel geïnteresseerd in uw mening.

Alvast bedankt voor uw deelname!

Kunt u aangeven op een schaal van 0-10 hoeveel u weet van het placebo-effect **op dit moment?**

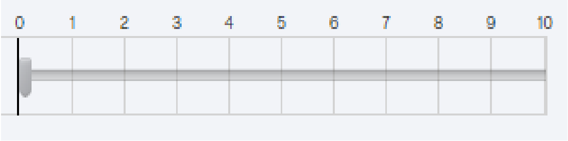


Een score van 0 betekent dat u hier nog <u>nooit</u> van gehoord heeft en een score van 10 betekent dat u <u>expert</u> bent.

---

## DE PLACEBO QUIZ

---

Geef hieronder aan welke stellingen u juist of onjuist vindt.

| | | Juist | Onjuist | *Correct antwoord* |
|---|---|---|---|---|
| 1. | Positieve verwachtingen kunnen een positief effect hebben op behandeluitkomsten | | | *Juist* |
| 2. | Placebo's worden gebruikt in wetenschappelijk onderzoek om nieuwe medicijnen te ontwikkelen | | | *Juist* |
| 3. | Een pil waar aspirine in zit heet een 'placebo' | | | *Onjuist* |
| 4. | Een placebo kan een symptoom zoals pijn verminderen | | | *Juist* |
| 5. | Gedachten kunnen gezondheid beïnvloeden | | | *Juist* |
| 6. | Vertrouwen in de arts en de behandeling dragen bij aan placebo-effecten | | | *Juist* |
| 7. | Placebo's kunnen een lichamelijke reactie veroorzaken | | | *Juist* |
| 8. | Als een patiënt weet dat hij/zij een placebo krijgt dan werkt het placebo-effect niet | | | *Onjuist* |
| 9. | Placebo's kunnen zorgen voor veranderingen in de hersenen (bijvoorbeeld het aanmaken van chemische stofjes) | | | *Juist* |
| 10. | Placebo-effecten komen alleen voor bij alternatieve geneesmiddelen (bijvoorbeeld acupunctuur of kruidenbehandeling) | | | *Onjuist* |
| 11. | De verpakking van de placebo (bijvoorbeeld de kleur van de pil) kan het effect hiervan beïnvloeden | | | *Juist* |
| 12. | Placebo's kunnen ook voor bijwerkingen zorgen | | | *Juist* |
| 13. | Placebo-effecten komen alleen voor in wetenschappelijk onderzoek | | | *Onjuist* |
| 14. | Het placebo-effect treedt alleen op bij psychische klachten zoals bijvoorbeeld stress | | | *Onjuist* |

# DE PLACEBO UITLEG

De Placebo Uitleg - Wij willen graag meer weten over de manier waarop het placebo-effect kan worden uitgelegd. Het is belangrijk dat de uitleg begrijpelijk en duidelijk is. Wij laten u verschillende manieren zien waarop behandelingen die gebruik maken van het placebo effect uitgelegd kunnen worden. We zijn benieuwd wat u hiervan vindt.

Beeld u in dat een behandelaar u de volgende uitleg geeft..

**Leren**
"Leren gebeurt niet alleen in het hoofd, maar ook (zonder dat u het door heeft) in het lichaam. Ons lichaam kan van medicatie iets leren: het onthoudt de lichamelijke reactie op medicatie. Dit heet **conditioneren**. Met een placebobehandeling maken we gebruik van deze aangeleerde reactie."

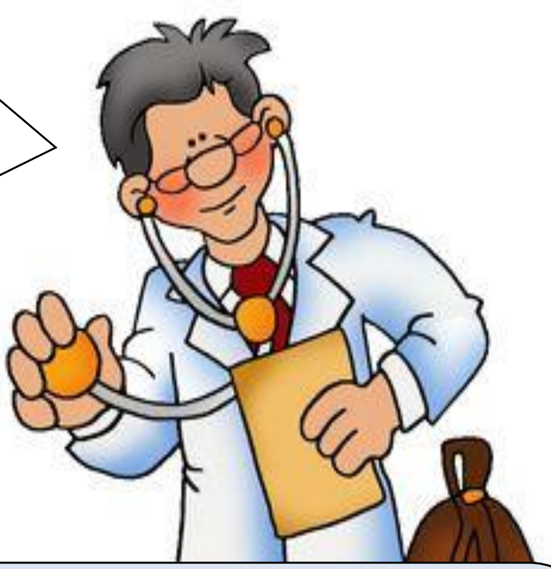

| | Sterk mee oneens | Oneens | Neutraal | Eens | Sterk mee eens |
|---|---|---|---|---|---|
| Denkt u dat een behandeling gebaseerd op deze uitleg **effectief** zal zijn? | O | O | O | O | O |
| Zou u op basis van deze uitleg **openstaan** voor deze behandeling? | O | O | O | O | O |

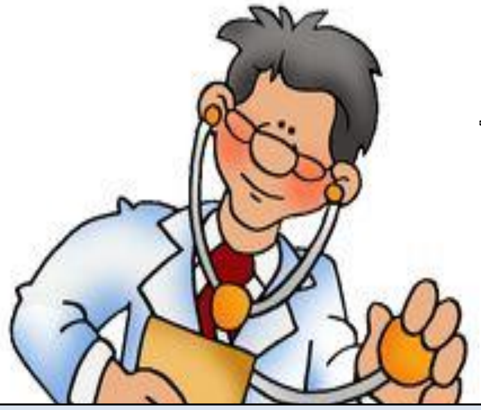

**Positieve verwachtingen**
"Over de werking van een medicijn of een behandeling kunnen we verwachtingen hebben. Als u een **positieve verwachting** heeft van een behandeling dan kan dit zorgen voor een beter behandel resultaat. U voelt zich dan niet alleen door de behandeling beter, maar ook door de positieve verwachting hiervan."

| | **Sterk mee oneens** | **Oneens** | **Neutraal** | **Eens** | **Sterk mee eens** |
|---|---|---|---|---|---|
| Denkt u dat een behandeling gebaseerd op deze uitleg **effectief** zal zijn? | O | O | O | O | O |
| Zou u op basis van deze uitleg **openstaan** voor deze behandeling? | O | O | O | O | O |

**Hersenen**
"Bij positieve verwachtingen over een behandeling maken de hersenen stofjes aan. Deze stofjes noemen we **neurotransmitters**, die ervoor zorgen dat u zich beter voelt. Na het nemen van een placebo komen er ook neurotransmitters vrij. Hier maken we bij een placebobehandeling onder andere gebruik van."

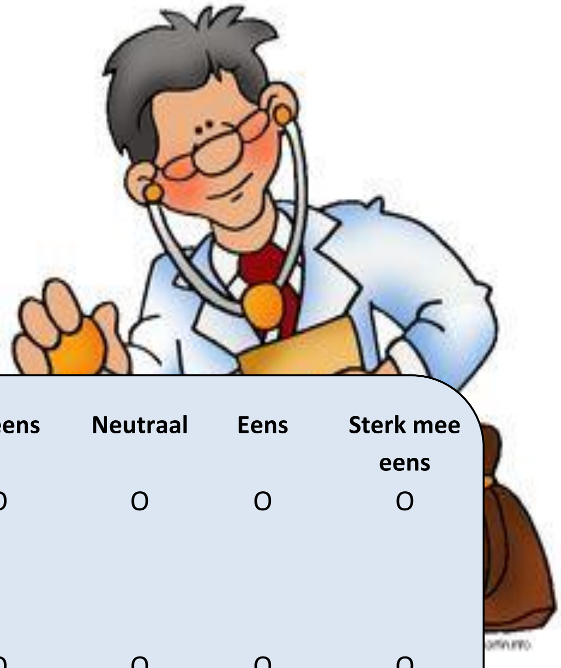

| | **Sterk mee oneens** | **Oneens** | **Neutraal** | **Eens** | **Sterk mee eens** |
|---|---|---|---|---|---|
| Denkt u dat een behandeling gebaseerd op deze uitleg **effectief** zal zijn? | O | O | O | O | O |
| Zou u op basis van deze uitleg **openstaan** voor deze behandeling? | O | O | O | O | O |

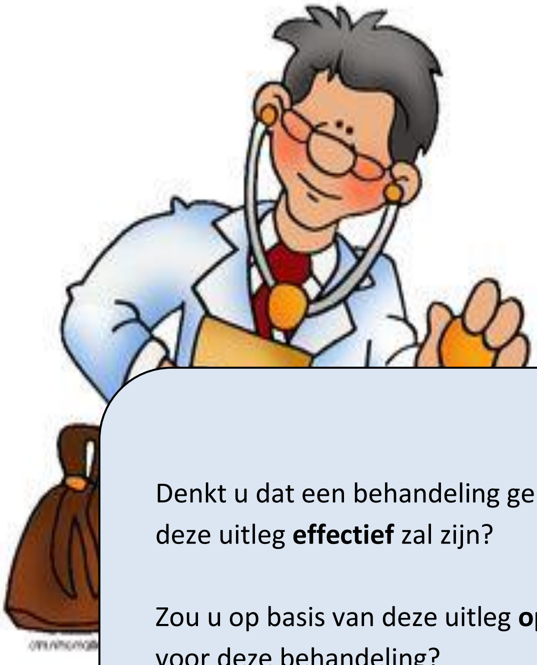

**Lichaam en geest**
“Dit is een behandeling gebaseerd op de samenwerking tussen **lichaam en geest**. De geest speelt een grote rol bij genezing. Een voorbeeld hiervan is het placebo-effect, wat laat zien dat een behandeling zonder actieve medicatie (placebopillen, zoals suikerpillen) ook voor een lichamelijke reactie kan zorgen.”

| | Sterk mee oneens | Oneens | Neutraal | Eens | Sterk mee eens |
|---|---|---|---|---|---|
| Denkt u dat een behandeling gebaseerd op deze uitleg **effectief** zal zijn? | O | O | O | O | O |
| Zou u op basis van deze uitleg **openstaan** voor deze behandeling? | O | O | O | O | O |

**Leren door observeren**
“Placebobehandelingen maken gebruik van de **ervaringen** die anderen hebben met een behandeling. Bijvoorbeeld, als u ziet of hoort dat andere mensen beter worden van een bepaalde behandeling, dan kan dit ook voor u helpen. Zo werkt het ook bij een placebobehandeling.”

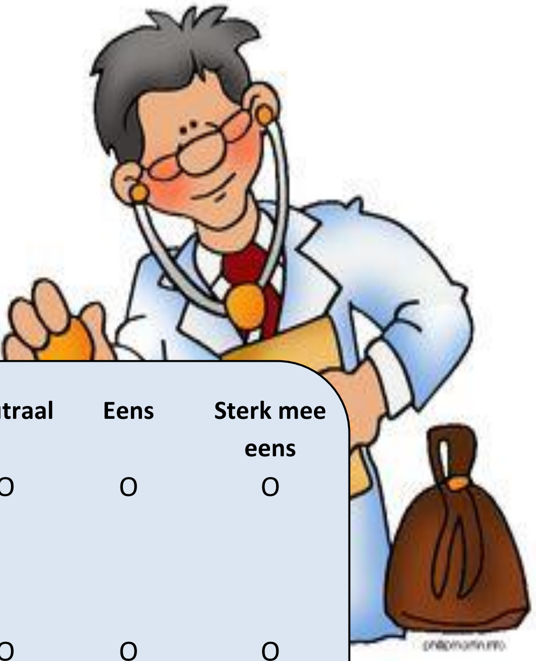

| | Sterk mee oneens | Oneens | Neutraal | Eens | Sterk mee eens |
|---|---|---|---|---|---|
| Denkt u dat een behandeling gebaseerd op deze uitleg **effectief** zal zijn? | O | O | O | O | O |
| Zou u op basis van deze uitleg **openstaan** voor deze behandeling? | O | O | O | O | O |

**Communicatie**

“Een goede band tussen de patiënt en de behandelaar kan een positieve invloed hebben op de behandeling. Het is bijvoorbeeld belangrijk dat de behandelaar het gevoel geeft **vertrouwen** te hebben in een behandeling. Bij placebobehandelingen maken we gebruik van deze vertrouwensband.”

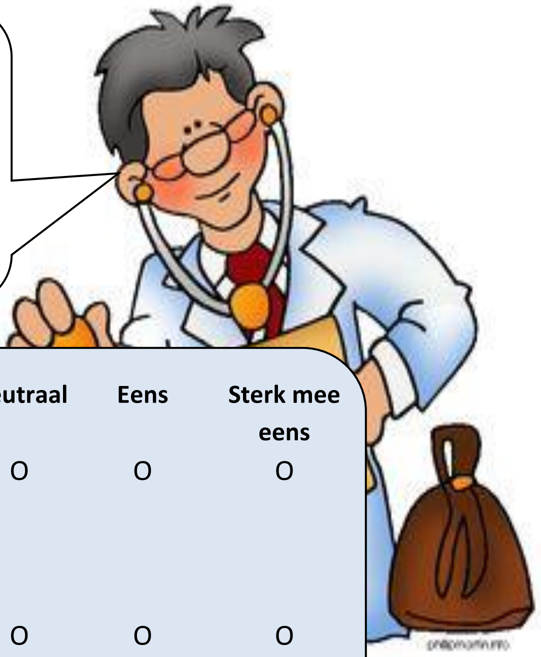

| | Sterk mee oneens | Oneens | Neutraal | Eens | Sterk mee eens |
|---|---|---|---|---|---|
| Denkt u dat een behandeling gebaseerd op deze uitleg **effectief** zal zijn? | O | O | O | O | O |
| Zou u op basis van deze uitleg **openstaan** voor deze behandeling? | O | O | O | O | O |

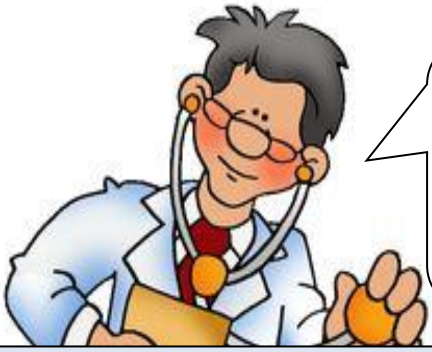

**Algemeen**

“Deze behandeling is gebaseerd op het placebo-effect. De behandeling kan u helpen, maar wij weten nog niet zeker hoe dit komt. Wij kunnen kijken of dit ook voor u van toepassing is.

| | Sterk mee oneens | Oneens | Neutraal | Eens | Sterk mee eens |
|---|---|---|---|---|---|
| Denkt u dat een behandeling gebaseerd op deze uitleg **effectief** zal zijn? | O | O | O | O | O |
| Zou u op basis van deze uitleg **openstaan** voor deze behandeling? | O | O | O | O | O |

**Eerlijkheid**

“In deze behandeling staat **eerlijkheid** voorop. Vroeger werd gedacht dat het placebo-effect alleen zou werken wanneer patiënten dachten dat zij eigenlijk ‘echte’ medicijnen kregen. Onderzoek heeft laten zien dat het placebo-effect ook werk als mensen weten dat het een placebo is. Dit willen wij graag in de behandeling toepassen.”

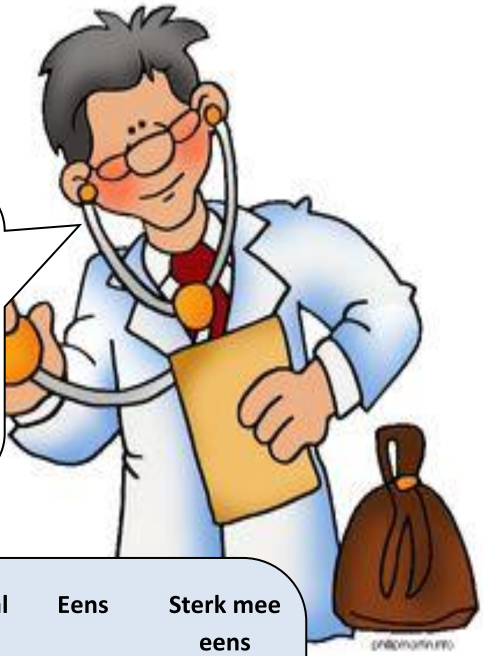

| | **Sterk mee oneens** | **Oneens** | **Neutraal** | **Eens** | **Sterk mee eens** |
|---|---|---|---|---|---|
| Denkt u dat een behandeling gebaseerd op deze uitleg **effectief** zal zijn? | O | O | O | O | O |
| Zou u op basis van deze uitleg **openstaan** voor deze behandeling? | O | O | O | O | O |

1. Alle uitspraken die u zojuist over het placebo-effect hebt gelezen zijn gebaseerd op wetenschappelijk onderzoek). **Kunt u aangeven op een schaal van 0-10 in hoeverre u deze uitleg zelf zou willen krijgen?**

**1) Leren**

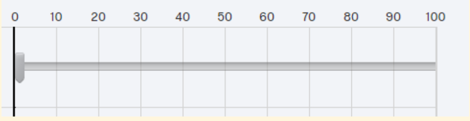


**2) Verwachtingen**

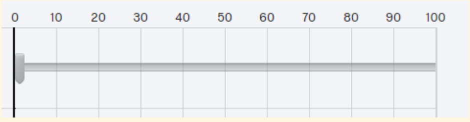


**3) Hersenen**

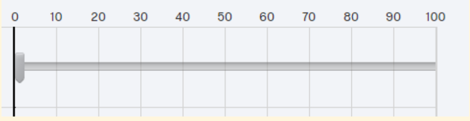


**4) Lichaam en geest**

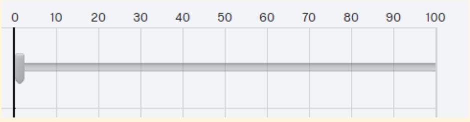


**5) Leren door observeren**

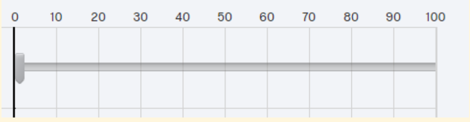


**6) Communicatie**

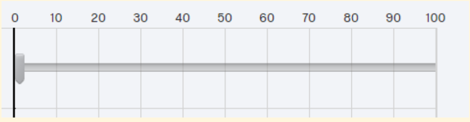


**7) Algemeen**

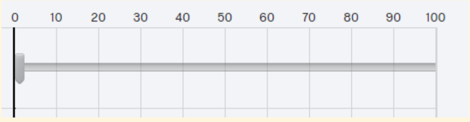


**8) Eerlijkheid**

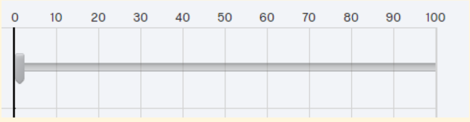


**Kunt u toelichten waarom een uitleg wel of niet uw voorkeur had?**

____________________________________________

**3.** Denkt u dat het **nuttig** is om placebo's in te zetten als behandeling?

| Sterk mee oneens | Oneens | Neutraal | Eens | Sterk mee eens |
|---|---|---|---|---|
| O | O | O | O | O |

| *Ik vind placebobehandeling acceptabel als...* | **Nooit** | **Bij psychologische klachten (bijv. stress of paniekaanvallen)** | **Bij milde gezondheids klachten (bijv. verkoudheid )** | **Bij een chronische ziekte (bijv reuma)** | **Bij een terminale ziekte (bijv kanker)** | **Anders…** |
|---|---|---|---|---|---|---|
| ... het gebruikt wordt **in combinatie met** een andere behandeling | | | | | | |
| … het gebruikt wordt **na langdurig medicatiegebruik** om te kijken of de patiënt zonder medicatie kan (afbouwen) | | | | | | |
| … er **geen andere behandeling** beschikbaar is | | | | | | |
| … het is aangetoond dat de placebo **hetzelfde effect** als het middel heeft, dan kan dit de hele behandeling vervangen | | | | | | |

***Hoe eerlijk moet de arts over placebo's zijn****?*

| | | | | | | |
|---|---|---|---|---|---|---|
| Als de arts denkt dat placebo's de beste oplossing zijn hoeft hij hier **niets over te zeggen** | | | | | | |
| De behandelaar hoeft alleen **achteraf** te zeggen dat het placebo's waren als het heeft geholpen | | | | | | |
| De arts moet hier **altijd** eerlijk over zijn**.** | | | | | | |
