# Supplementary material for: Explaining placebo effects in an online survey study: Does ‘Pavlov’ ring a bell?
Source: PLoS One. 2021 Mar 11;16(3):e0247103. doi: 10.1371/journal.pone.0247103 (PMC7951811; doi:10.1371/journal.pone.0247103)
Supplement: S5 File — (PDF) [file pone.0247103.s005.pdf]

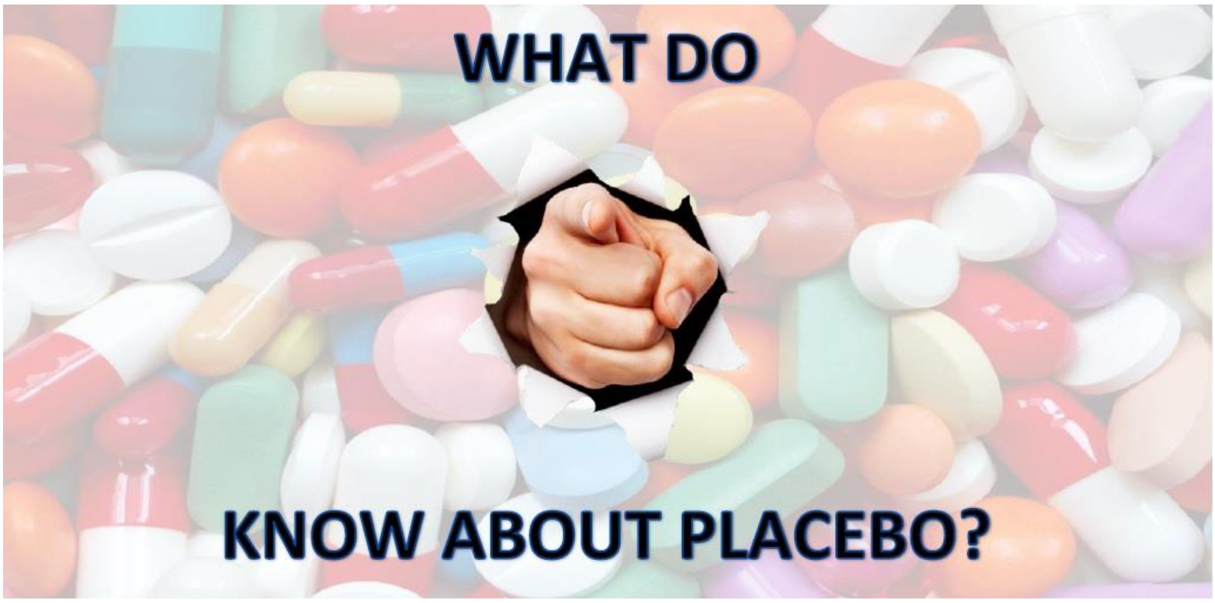

Placebos
Placebos are often used in scientific research. These are treatments that do not consist of active ingredients. However, people can still feel better after taking a placebo. This is called the placebo effect.

Please answer all questions from your own point of view and based on what you know about placebos. It is important that your responses are as honest as possible. There are no right or wrong answers, we are only interested in your opinion.

Thank you for your participation!

Please indicate on a scale from 0-10 how much you know about the placebo-effect **at this moment?**

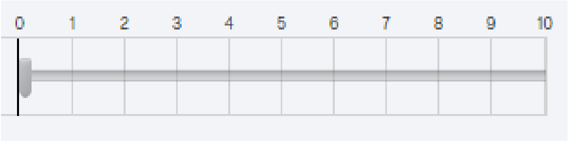

A score of 0 indicates that u have <u>never</u> heard of the placebo effect and a score of 10 indicates that you are an <u>expert.</u>

THE PLACEBO QUIZ

Please indicate which statements you believe are true or false.

		True	False	*Correct Answer*
1.	Positive expectations can have a positive effect on health			*True*
2.	Placebos are used to develop new drugs			*True*
3.	A pill that contains aspirin is called a 'placebo'			*False*
4.	A placebo can reduce symptoms such as pain			*True*
5.	Thoughts can affect your health			*True*
6.	Trust in the physician and the prescribed treatment can add to placebo effects			*True*
7.	Placebos can induce a physical reaction			*True*
8.	Placebo effects do not work when a person knows he or she is taking a placebo			*False*
9.	Placebos can cause for a changes in the brain (such as producing chemical substances)			*True*
10.	Placebo effects only occur in alternative medicine (such as acupuncture or herbal medicines)			*False*
11.	The packaging of a placebo (i.e. color of the pill) can influence its effects			*True*
12.	Placebos can also cause for adverse effects			*True*
13.	Placebo effects only occur in scientific research			*False*
14.	Placebo effects are only present in psychological complaints (such as stress)			*False*

THE PLACEBO EXPLANATION

The Placebo Explanation – We want to know more about the different ways of explaining the placebo effect. It is important that the explanation is easy to understand and clear. We will show you different ways how treatments that make use of placebo effects can be explained. We would like to hear your opinion about each explanation.

Imagine that a physician gives you the following explanation..

Learning
"Learning doesn't only happen in the brain, but also in the body even when you are not aware of this. Our body can learn from taking medication; it remembers what happens and what chemicals are produced. This is called conditioning. In this placebo treatment we will make use of the body's learned response to reduce pain."

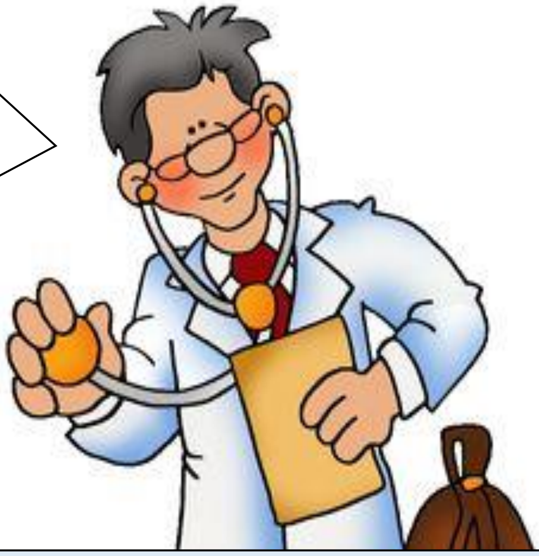

	**Strongly disagree**	**Disagree**	**Neutral**	**Agree**	**Strongly agree**
Do you believe that a treatment based on this explanation will be **effective**?	O	O	O	O	O
Would you be **willing to receive** this treatment based on this explanation?	O	O	O	O	O

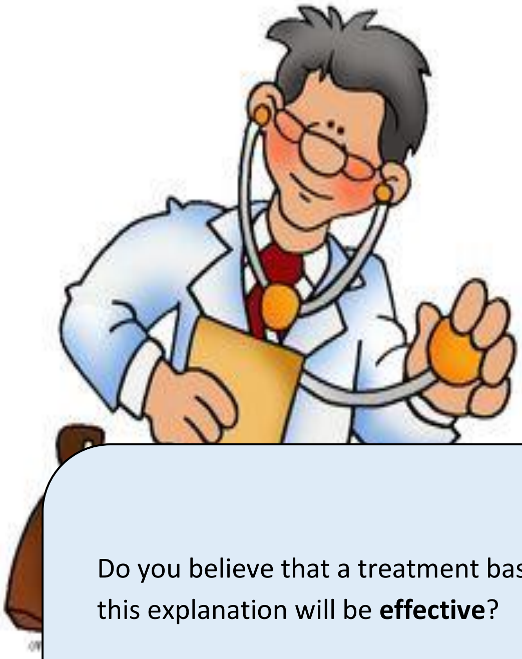

Positive expectations

"You can have expectations about a treatment and its outcomes. If you have positive expectations, this may cause for a more positive treatment outcome and it may also affect your pain experience in this present study. You may not only feel better from the treatment itself, but also because you expect to feel better."

	Strongly disagree	Disagree	Neutral	Agree	Strongly agree
Do you believe that a treatment based on this explanation will be **effective**?	O	O	O	O	O
Would you be **willing to receive** this treatment based on this explanation?	O	O	O	O	O

Brain

"When you have positive expectations, the brain produces chemicals. These chemical substances are called neurotransmitters and can make you feel better. Placebo's can also trigger the release of neurotransmitters. In this treatment we will be making use of this chemical process to reduce pain."

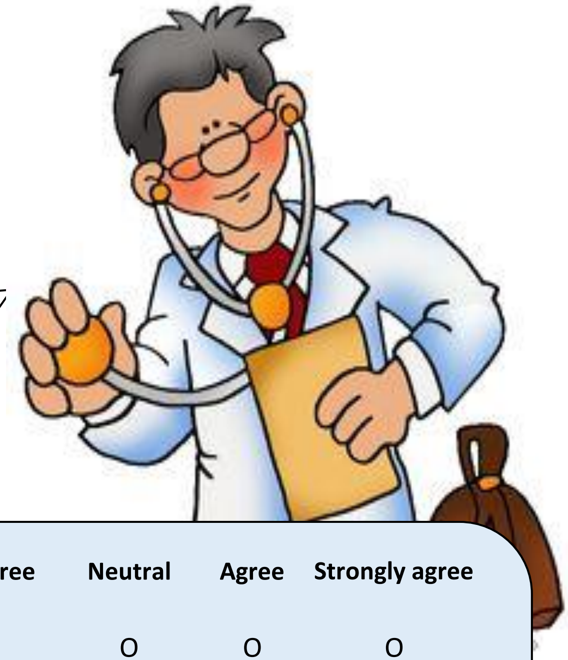

	Strongly disagree	Disagree	Neutral	Agree	Strongly agree
Do you believe that a treatment based on this explanation will be **effective**?	O	O	O	O	O
Would you be **willing to receive** this treatment based on this explanation?	O	O	O	O	O

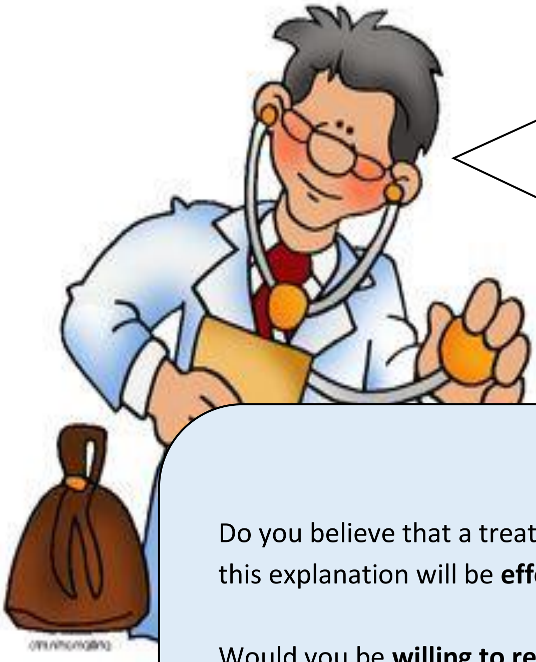

Mind and body
"This treatment is based on the interaction between **mind and body**. The mind plays a big role in healing. The placebo effect is an example of this, because it shows that treatments without active medication (placebo pills, like sugar pills) can also evoke a physical reaction."

	Strongly disagree	Disagree	Neutral	Agree	Strongly agree
Do you believe that a treatment based on this explanation will be **effective**?	O	O	O	O	O
Would you be **willing to receive** this treatment based on this explanation?	O	O	O	O	O

Observational learning
"Placebo treatment make use of the **experiences** that others have with treatments. Seeing or hearing others benefit from treatment, may cause you to feel better after this treatment as well."

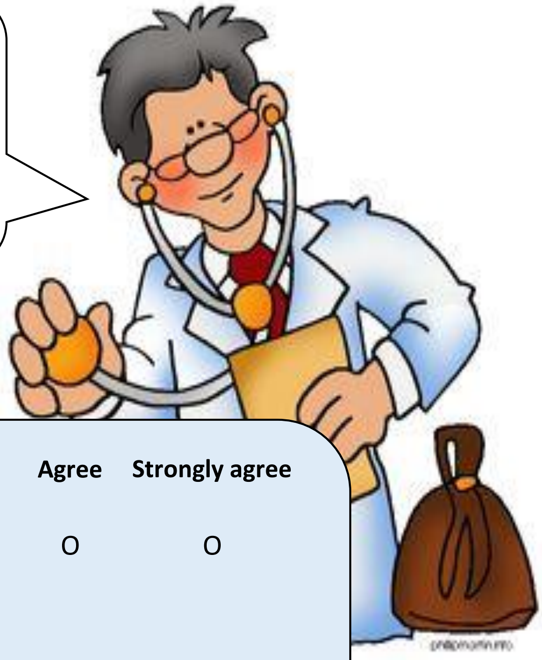

	Strongly disagree	Disagree	Neutral	Agree	Strongly agree
Do you believe that a treatment based on this explanation will be **effective**?	O	O	O	O	O
Would you be **willing to receive** this treatment based on this explanation?	O	O	O	O	O

Communication
"A good relationship with your doctor can have a positive impact on treatment outcomes. For example, it is important that the doctor makes you feel that he or she **trusts** the prescribed treatment. Placebo treatments are based on the importance of this relationship of trust."

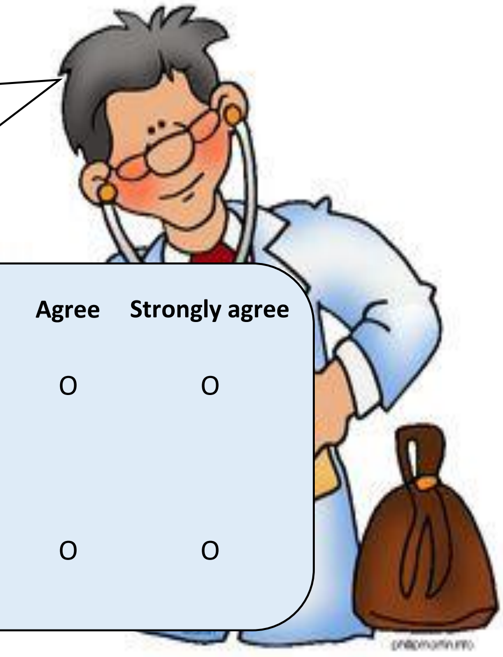

	Strongly disagree	Disagree	Neutral	Agree	Strongly agree
Do you believe that a treatment based on this explanation will be **effective**?	O	O	O	O	O
Would you be **willing to receive** this treatment based on this explanation?	O	O	O	O	O

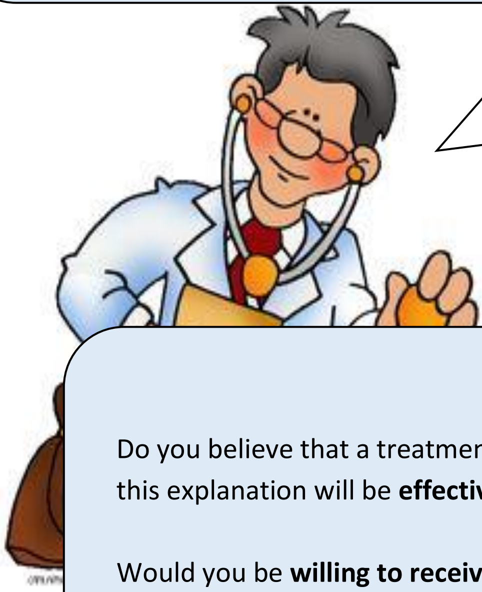

Neutral
This treatment is based on the placebo effect. This treatment may help you, but we are still researching how this exactly works. We can find out if this works for you too."

	Strongly disagree	Disagree	Neutral	Agree	Strongly agree
Do you believe that a treatment based on this explanation will be **effective**?	O	O	O	O	O
Would you be **willing to receive** this treatment based on this explanation?	O	O	O	O	O

Transparency
"**Honesty** is the most important factor in this treatment. Previously, it was thought that deception was necessary in order for a placebo effect to occur. However, research has demonstrated that placebo effects are also present when people know they are taking a placebo. We would like to apply this in this treatment."."

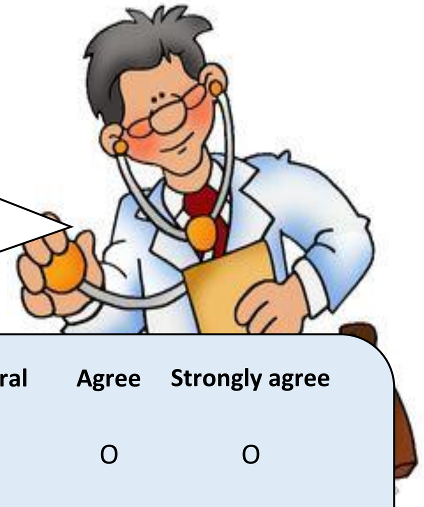

	Strongly disagree	Disagree	Neutral	Agree	Strongly agree
Do you believe that a treatment based on this explanation will be **effective**?	O	O	O	O	O
Would you be **willing to receive** this treatment based on this explanation?	O	O	O	O	O

1. All explanations about placebo effects that you have read are based on scientific research. **Please indicate on a scale from 0-100 how much you want to receive the corresponding explanation** (all explanations were presented together with the sliders below)?

1) Learning

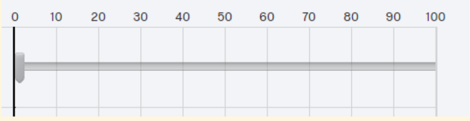

2) Positive expectations

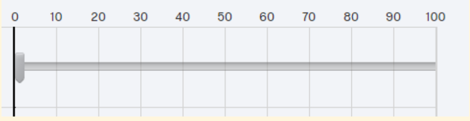

3) Brain

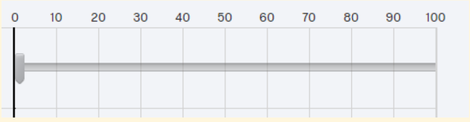

4) Mind and body

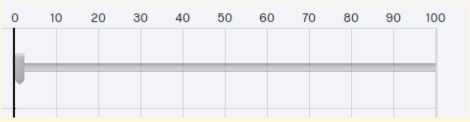

5) Observational learning

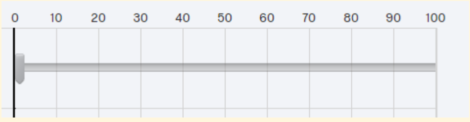

6) Communication

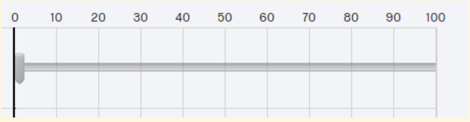

7) Neutral

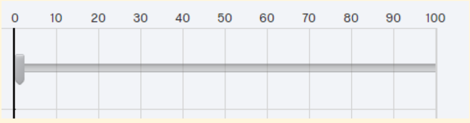

8) Transparency

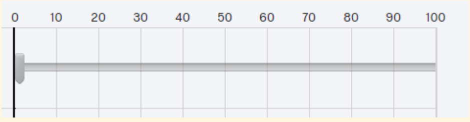

Please indicate why you preferred certain explanations and why you didnt prefer others.

____

3. Do you believe it is **useful** to use placebos in treatments?

**Sterk mee oneens**	**Oneens**	**Neutraal**	**Eens**	**Sterk mee eens**
O	O	O	O	O

**Placebo treatment is acceptable when..**	**Never**	**Psychological complaints (e.g. stress or anxiety)**	**Mild health complaints (e.g. a cold)**	**Chronic disease (e.g. arthritis)**	**Terminal disease (e.g. cancer)**	**Alwa ys**	**Other…**
... it is **combined with** another medical treatment.							
…it is prescribed after **a long period** of medication to assess whether medication can be tapered off.							
… there is no **alternative treatment** is available.							
… placebos can **substitute** the entire treatment when it is proven that placebo effects are not inferior to actual treatment effects.							

How honest should a physician be about prescribing placebos?	Never	Psychological complaints (e.g. stress or anxiety)	Mild health complaints (e.g. a cold)	Chronic disease (e.g. arthritis)	Terminal disease (e.g. cancer)	Always	Other…
If the physician assumes that it will benefit the patient, he or she **does not have to disclose placebo use.**							
The physician only has to disclose placebo use **when it worked.**							
The physician should **always** be honest about placebo use							
