# Supplementary material for: Explaining placebo effects in an online survey study: Does ‘Pavlov’ ring a bell?
Source: PLoS One. 2021 Mar 11;16(3):e0247103. doi: 10.1371/journal.pone.0247103 (PMC7951811; doi:10.1371/journal.pone.0247103)
Supplement: Attachment — Submitted filename: PONE-D-20-16802_reviewer.docx [file pone.0247103.s006.docx]

Thank you for the opportunity to review this manuscript. This was a cross-sectional, survey-based study from Smits and colleagues at Leiden University in the Netherlands. The overall purpose was to characterize preference for various placebo information strategies for optimization in clinical practice. Specifically, the authors aimed to (1) examine current knowledge about placebo effects, (2) rate different types of explanations about placebo effects and their mechanisms based on preference, perceived efficacy, and willingness participate in a treatment based on a given placebo explanation, (3) extend prior work examining attitudes and acceptability towards placebos in treatment settings, (4) examine the underlying factor structure of their placebo explanations scale, and (5) examine the extent to which factors associated with placebo effects explained variance in explanation preferences.

Out of 444 total participants, 377 completed the survey. The primary findings are as follows:

(1) **Placebo knowledge:** Mean correct score of 81.1% on the placebo knowledge quiz with the lowest score observed for an item concerning whether deception is required in order to induce placebo effects (22.5 % correct)
(2) **Placebo knowledge:** 13.6% of variance in placebo knowledge was explained in the model. Education was the only significant predictor.
(3) **Differences in placebo explanations**

**Preference:** Participants differed in terms of preference for explanations with those based on brain mechanisms and expectations showing higher preference than others. The neutral explanation had significantly lower preference than the others.

**Perceived efficacy:** No differences

**Willingness to participate:** No differences

(4) **Predictors of placebo explanations**

**Preference:** Age, education, optimism, trait anxiety, and placebo knowledge

**Perceived efficacy:** optimism, trait anxiety, placebo knowledge and education

**Willingness to participate:** trait anxiety and general attitudes towards medication

(5) **Factor structure**

**Preference:** 1 factor explaining 36.24% of the variance

**Perceived efficacy:** 2 factors, 1 explaining 19.68% of the variance and 2 explaining 15.41% of the variance

**Willingness to participate:** 2 factors, 1 explaining 22.74% of the variance and 2 explaining 15.81% of the variance

(6) **Placebo attitudes and acceptability:**

Overall, I thought the study design was interesting and believe that this manuscript would add a unique perspective to the broader literature on placebo effects. However, the statistical analysis, results, and discussion sections in particular could benefit from more development. Thus, I would like to see your detailed response to my below comments before giving this manuscript any further consideration for publication.

**Introduction-**

**123-139 –** Consider making the order of your aims and results consistent for the reader. For instance, the aim to explore attitudes appears third in the list of aims whereas these data are presented last in the results section.

**134-136 –** Can you provide a stronger rationale for exploring the factor structure of your preference, perceived efficacy, and willingness to participate scales? How would this information benefit clinicians who want to use placebos in practice? As of now, I am having trouble understanding what this information adds to the overall manuscript and it seems more appropriate for a separate study of psychometric properties of their instrument (e.g., factorial validity)

**Methods –**

**166-168** -You designed your study to control for order effects when collecting data on placebo explanations. Can you describe this a bit more? How many possible orders were available to a given participant? Did you confirm whether or not order had an effect on your results (e.g., does entering order as a covariate in your model affect the results)?

**213-239-** The investigators have used several standardized scales to help characterize the participant sample (i.e., Life Orientation Test-Revised, Revised NEO Personality Inventory, Spielberger State Trait Anxiety Inventory, General Attitudes towards Medication Survey). If available, please consider providing norms for each of these scales to help the reader contextualize whether the study sample is above or below average on each of these scales.

**240-** Can the authors comment on the statistical power of their study? Was an *a priori* power analysis conducted for any of the aims?

**248-260-** It is unclear to me why a repeated-measures model is being used here. Wouldn’t a one-way ANOVA/ANCOVA be sufficient since the overall design of the study did not involve multiple timepoints?

**Results -**

**301-307 –** Please provide the results for the overall regression model. Was the overall model significant? This is critical for interpreting the rest of your findings. In other words, if the overall model was not significant, observing individual predictors that were significant (e.g., education) does not really matter.

**328-381-** The authors describe results of three different regression models presumably examining predictors of preference, perceived efficacy, and willingness to participate, but these models are not described in the statistical analysis section. What kind of regression model was used? How was the dependent variable score treated? For each model, was the score a sum total from the various items used to characterize preference, perceived efficacy, and willingness to participate? This will definitely need more detail.

**329-338-** Please provide the results for the overall regression model in addition to your summary of individual predictors. Was the overall model significant?

**351-362-** Please provide the results for the overall regression model in addition to your summary of individual predictors. Was the overall model significant?

**372-381-** Please provide the results for the overall regression model in addition to your summary of individual predictors. Was the overall model significant?

**Discussion-**

**444-446-** The investigator appears to interpret “Placebos can also cause side effects” as knowledge about nocebo effects. Indeed, information about nocebo effect knowledge is very important, but I am not entirely convinced that is what they measured with this item and would urge the authors to exercise some caution with referring to it as such. How do we know the respondents are assigning a negative connotation to the term “side effects”? It is unfortunate that this was not spelled out more clearly for study participants (e.g., Placebos can also cause negative or harmful effects).

**444-447 –** There’s hardly any discussion of the regression model that explored predictors of placebo quiz performance and found 13.6% variance explained. Was the amount of variance explained lower or higher than the authors expected? What other factors could account for the large amount of variance that was not explained by the model?

**456-474 –** I found the exploration and discussion of the underlying factor structure to be rather distracting and I am not sure this adds much value to the paper. In your revisions, please either make this more apparent to the reader or consider removing altogether.

**479-480** – “Although the sample was rather large (377 completers)…”. Relative to which specific studies?

**486-489 –** Despite listing this as one of their aims, there’s also very little discussion of how exactly their results have extended prior work examining attitudes and acceptability towards placebos in treatment settings. I think this could be a separate paragraph rather than squeezed in as an afterthought in it’s present place in the manuscript.

**Limitations/Future directions section** –

The discussion of the study limitations is pretty thin. Please add a paragraph or two on the potential shortcomings of this study to help the reader contextualize the findings. A few things that come to mind are:

(1) The cross-sectional nature of the study (e.g., what if attitudes change over time as new experiences occur (e.g., clinical trial participation, patient-provider interactions?). This is especially relevant considering how young your sample is.
(2) How might various populations with chronic mental or physical health conditions benefit from this information? Is the present data sufficient for these groups or is more data directly collected from patient populations needed?
(3) Is there anything to be learned from applying this research question/approach to nocebo effects and more thoroughly characterizing knowledge of nocebo effects than what was done here?
(4) Just because a participant prefers a given strategy does not necessarily mean this translates to the largest placebo effects. This of course is an empirical question that could be tested in an experimental design and merits some thought/discussion about how such a study could be conducted.

To be clear, these are just some potential ideas worth considering and I’m not saying that each of these needs to be added as limitations. Rather, I would like to see you give some thoughtful consideration to what you think are the primary limitations and discuss them accordingly.
